# Using smartphones to decrease substance use via self-monitoring and recovery support: study protocol for a randomized control trial

**DOI:** 10.1186/s13063-017-2096-z

**Published:** 2017-08-10

**Authors:** Christy K Scott, Michael L. Dennis, David H. Gustafson

**Affiliations:** 1 Lighthouse Institute, Chestnut Health Systems, 221 W. Walton, Chicago, IL 60610 USA; 2 Lighthouse Institute, Chestnut Health Systems, 448 Wylie Dr, Normal, IL 61761 USA; 3 Center for Health Enhancement Systems Studies, Industrial and Systems Engineering Department, University of Wisconsin–Madison, Madison, WI 53706 USA

**Keywords:** Smartphone, Ecological momentary assessment, Ecological momentary intervention, Substance use disorder, Recovery support, Technology, eHealth, mHealth

## Abstract

**Background:**

Alcohol abuse, other substance use disorders, and risk behaviors associated with the human immunodeficiency virus (HIV) represent three of the top 10 modifiable causes of mortality in the US. Despite evidence that continuing care is effective in sustaining recovery from substance use disorders and associated behaviors, patients rarely receive it. Smartphone applications (apps) have been effective in delivering continuing care to patients almost anywhere and anytime. This study tests the effectiveness of two components of such apps: ongoing self-monitoring through Ecological Momentary Assessments (EMAs) and immediate recovery support through Ecological Momentary Interventions (EMIs).

**Methods/design:**

The target population, adults enrolled in substance use disorder treatment (*n* = 400), are being recruited from treatment centers in Chicago and randomly assigned to one of four conditions upon discharge in a 2 × 2 factorial design. Participants receive (1) EMAs only, (2) EMIs only, (3) combined EMAs + EMIs, or (4) a control condition without EMA or EMI for 6 months. People in the experimental conditions receive smartphones with the apps (EMA and/or EMI) specific to their condition. Phones alert participants in the EMA and EMA + EMI conditions at five random times per day and present participants with questions about people, places, activities, and feelings that they experienced in the past 30 min and whether these factors make them want to use substances, support their recovery, or have no impact. Those in the EMI and EMA + EMI conditions have continual access to a suite of support services. In the EMA + EMI condition, participants are prompted to use the EMI(s) when responses to the EMA(s) indicate risk. All groups have access to recovery support as usual. The primary outcome is days of abstinence from alcohol and other drugs. Secondary outcomes are number of HIV risk behaviors and whether abstinence mediates the effects of EMA, EMI, or EMA + EMI on HIV risk behaviors.

**Discussion:**

This project will enable the field to learn more about the effects of EMAs and EMIs on substance use disorders and HIV risk behaviors, an understanding that could potentially make treatment and recovery more effective and more widely accessible.

**Trial registration:**

ClinicalTrials.gov, ID: NCT02132481. Registered on 5 May 2014.

**Electronic supplementary material:**

The online version of this article (doi:10.1186/s13063-017-2096-z) contains supplementary material, which is available to authorized users.

## Background

Cost estimates of 60 major illnesses place alcohol use disorders as the second most costly health problem and other drug use disorders as the seventh most costly health problem in the US [1]. Both conditions relate to higher rates of risk behaviors associated with the human immunodeficiency virus (HIV). The three conditions represent three of the top 10 modifiable behavioral causes of death in the US. [2].

Studies indicate that effective strategies for managing substance use disorders (SUDs) must address their chronic and cyclical nature [3–7]. Untreated SUDs create circumstances, such as needle use and trading sex for drugs, that increase HIV risk [8], disease burden, and mortality [9, 10]. Although continuing care with monitoring has been associated with better outcomes for SUDs [11], as it has been for other chronic conditions [12], patients with SUDs rarely receive it [12, 13]. Mobile technology can provide tools for ongoing monitoring and assessment and access to recovery support interventions anytime and anywhere, thus offering the potential to radically improve the odds of a patient’s sustained recovery.

In recent decades, information and communication technologies (ICTs) have provided a number of options for self-monitoring, self-management, and self-initiated accessibility to care, including Internet programs [14, 15], telephone support [16, 17], telephone continuing care [18–21], text messaging [22, 23], and multiservice interventions [24]. Recently, attention has focused on using smartphones as a platform for providing multiple services. A recent meta-analysis [25] identified six smartphone applications (apps), four of which had been rigorously evaluated. Two demonstrated an association with reduced alcohol use, including the app used in the research described below.

### Using Ecological Momentary Assessments (EMAs) to self-monitor and change behavior

Fundamental to effectively self-managing recovery is one’s ability to monitor the interplay between one’s internal states (e.g., feelings, cravings) and external factors (e.g., people, places, activities) that have been shown to influence recovery. EMAs help people to self-monitor behaviors at the time and in the context in which they occur. Self-monitoring asks patients to note internal and external factors that take place with a target behavior such as substance use. Behavioral self-monitoring has been employed for several decades both as a means of studying behavior and as a component of therapeutic interventions [26]. In behavioral therapy, it has been used both as an assessment and an intervention to change behavior. Self-monitoring is often considered a key component of self-management [27] because it can give patients insight into the dynamic relationship between these factors and the target behavior [28]. Problem behaviors, such as SUDs, may be affected by multiple factors that may not be obvious to the individual but are revealed through frequent self-monitoring. The frequency and duration of scheduled EMAs can influence the impact of self-monitoring on behavior change. For example, Kauer and colleagues [29] demonstrated that increasing the rate of monitoring from one to six times a day was related to increased emotional self-awareness and subsequent reductions in depressive symptoms. Frequent self-monitoring has helped curtail risky behaviors under theories related to mindfulness and relapse prevention [28, 30–32].

### Using EMA responses to predict risk

In addition to monitoring in real time the factors related to target behaviors, EMAs have been used to calculate the risk for new episodes of the target behavior—e.g., in schizophrenia [33–35]. Chih et al. [36] found that relapse to substance use could be predicted using information from a weekly survey on use in the prior week and risk and protective factors. In a pilot test with adolescents, Dennis et al. [37] developed a model of predictive analytics using three categories of risk based on the EMA used in this study. The predictive value of this model will be prospectively tested in the current study.

### Using Ecological Momentary Interventions (EMIs) to provide immediate intervention

Ecological Momentary Interventions (EMIs) are provided during people’s everyday lives (i.e., in real time) anywhere they spend time (i.e., the real world) [38, 39]. EMIs vary in their level of human interactivity [40]. At the high end of the range, patients may access a live therapist for a full therapy session or response to an emergency [41] or get help from peers or family [42, 43]. At the low end of the range, interventions may rely on educational components, breathing exercises, and relaxation recordings [44]. A common limitation of previous studies is that EMIs are self-initiated; and even though EMIs are often available 24/7, individuals do not necessarily access them in response to risky situations [45, 46].

### Using EMA responses to prompt EMI use

One response to these issues is to use EMA responses to tailor the delivery of EMIs rather than rely on the individual to initiate EMI use. One previously used method of tailoring involves having participants preidentify high-risk times and scheduling the delivery of EMIs during these risky times, as has been done in some smoking cessation studies [47–50]. Although this strategy works well when target behaviors are highly predictable, many are not. An alternative is to use EMA responses that indicate risk to trigger the automatic delivery of EMIs. This tailoring method requires that the circumstances that trigger the delivery of the intervention be clearly defined and measurable. Because substance use and many high-risk behaviors are discrete events with identifiable antecedents and consequences, they are ideal for integrating EMAs and EMIs.

This study will advance the field by using data from multiple daily EMAs to provide immediate feedback to participants about their risk of use and interventions (EMIs) designed to reduce risk and increase abstinence [51, 52]. Our team’s pilot study [37] demonstrated that using Addiction – Comprehensive Health Enhancement Support System (A-CHESS) within an hour of the EMA was associated with reducing by half the risk for use in the next 7 days. This finding will be prospectively tested in the current study with a factorial design. One important empirical question the study addresses is whether links between EMAs and EMIs improve outcomes beyond the effects of EMAs and EMIs alone. The primary goal of the trial is to examine the effect of combining, over 6 months, frequent self-monitoring (via EMAs) with immediate interventions (via EMIs) on days of abstinence from drugs and alcohol and, secondarily, number of HIV risk behaviors.

## Methods/design

### Aims and hypotheses

The aims of this randomized controlled trial (RCT) are to (1) test the effect of EMAs, EMIs, and EMAs + EMIs on days of abstinence from alcohol and other drugs, (2) test the effect of EMAs, EMIs, and EMAs + EMIs on HIV risk behaviors, and (3) evaluate the extent to which changes in days of abstinence mediate the effect of EMAs, EMIs, and EMAs + EMIs on HIV risk behaviors. The primary hypothesis is that (H1) participants assigned to (H1a) EMAs, (H1b) EMIs, and (H1c) EMAs + EMIs will have, compared with control-group participants, more days of abstinence from drugs and alcohol in the 6 months post discharge from treatment. The two secondary hypotheses are that (H2) participants assigned to EMAs, EMIs, and EMAs + EMIs will have, compared with control-group participants, fewer HIV risk behaviors in the 6 months post discharge, and that (H3) days abstinent at 3 months post discharge will mediate the effects of EMAs, EMIs, and EMAs + EMIs on HIV risk behavior at 6 months post discharge.

### Design

Participants are randomly assigned in a 2 × 2 factorial design to receive EMAs only, EMIs only, combined EMAs + EMIs, or recovery support as usual (control). Participants in the three EMA/EMI groups receive a smartphone and training. Individuals in the two groups receiving EMIs can initiate at any time a suite of interventions designed to support recovery. In the EMAs + EMIs group, participants receive feedback directly after they complete each EMA; risky EMA responses are used to encourage EMI use.

### Participants

Men and women are eligible if they: (1) are 18 years old or older, (2) met criteria for SUDs in the year prior to treatment intake, (3) currently live in Chicago, (4) can communicate in English, and (5) are cognitively able to provide informed consent. Individuals are ineligible if they: (1) currently live outside Chicago or plan to live outside of Chicago during the 6 months of the study, (2) expect to be in jail, prison, or another setting that would prevent the use of smartphones, (3) are unable to use a smartphone because of a disability or health condition, (4) are unwilling to learn to use a smartphone or to complete a survey using a smartphone, (5) are admitted to a treatment program that provides intensive services post discharge, (6) have a recovery coach and have been in contact with the recovery coach in the last 30 days, (7) fail the Short Blessed cognitive impairment test [53], and (8) have ever been diagnosed with, or told by a physician that they have, schizophrenia and/or are bi-polar.

### Interventions

Participants in all four randomization groups receive recovery support as usual (RSAU), which includes relapse-prevention training. All participants also visit the research office twice during the first month of the intervention period and at months 3 and 6 after enrollment to complete surveys and for urine monitoring. Participants in the three treatment groups receive an Android phone after randomization. Table 1 shows the intervention components by group; the interventions are described below.

**Table 1:** Intervention components

Component	Randomization group			
	Control	EMA only	EMI only	EMA + EMI
Recovery support as usual, including relapse-prevention training	X	X	X	X
Research office visits twice during month 1	X	X	X	X
Urine monitoring	X	X	X	X
Android phone		X	X	X
EMA training		X		X
5 EMA prompts daily over 16 h/day		X		X
EMI training			X	X
Specific EMI recommendations				X

*EMA* Ecological Momentary Assessment, *EMI* Ecological Momentary Intervention

### Recovery support as usual (control group)

Standard discharge practice is to provide a recovery plan and relevant referrals in the community. A recent RCT that examined RSAU for patients discharged from one of the main recruitment sites indicated that 37% of clients attended self-help in the community, 41% participated in substance-free structured activities, and about 14% returned to treatment within 6 months [6]. Participants also receive relapse-prevention training, during which they review how people, places, things, and feelings can support their recovery and/or operate as triggers.

### EMAs

Participants in the EMA-only condition receive a 2-h training on how to use the phone, how EMAs can help with self-monitoring, and procedures for completing the EMAs. During participants’ two visits to the research office in month 1, research staff check participants’ proficiency in using the phone, offering help as necessary. EMAs are delivered five times a day within a range of 16 h established by the participant. EMAs consist of the same set of 28 questions with possible answers. Although the questions and answers remain the same, the order of the questions and the order of the answers vary randomly. Questions ask participants to record their recent substance use, HIV risk behaviors (e.g., needle use, unprotected sex), and exposure in the last 30 min to internal and external protective and risk factors (people, places, activities, and feelings), and then to rate the extent to which these factors support their recovery, make them want to use drugs or alcohol, or have no impact. Participants receive a “thank-you” when they complete the EMA. Each EMA takes 2 to 3 min to complete.

### EMIs

Participants in the EMI-only condition receive a 2-h training on how to use the phone, how EMIs relate to relapse prevention or HIV risk reduction, and how to access each EMI. Like those in the EMA condition, EMI-group participants have their proficiency with the phone checked during their two research office visits in month 1, getting help if needed.

EMIs come from A-CHESS, a smartphone system designed to provide continuing care for patients with alcohol use disorders [45]. A-CHESS was tested in a randomized clinical trial and found to reduce risky drinking days [45]. The system, which is informed by Marlatt’s relapse prevention model [30], includes information about addiction and recovery, reminders about motivators and healthy coping mechanisms, healthy activities sent through alerts and shared calendars, distractions from craving and negative affect through games and social networking, personal stories about others in recovery, and social supports. Social support is important for managing several chronic diseases [54, 55] and is an integral part of A-CHESS. The goal of the social support tools is to develop for each user a network that can help when the user feels at risk of a lapse [56, 57]. A-CHESS social supports are discussion groups (with use guidelines and monitoring), the support team [58], and text messaging [59–61].

Although A-CHESS reduced risky drinking days among participants who had it versus the control group, the clinical trial raised issues that the design of the current study will allow us to examine. For example, participants rated themselves on protective and risk factors weekly, which made the ratings subject to recall bias; opportunities to intervene in the moment of greatest need were lost if users did not initiate use of A-CHESS in the moment; and “teachable moments”—when participants might have consciously linked risk factors to their desire for or actual use of alcohol—may have been lost.

### EMAs + EMIs

Participants in the EMA + EMI condition receive the interventions described above, with EMI training taking place a week after EMA training. In pilot studies, we found that participants could easily learn to operate the phone and complete EMAs one week and then integrate EMIs the next week, but doing all the training at once was too much. Like participants in the EMA group, participants in the combined group receive a thank-you when they complete an EMA, but in addition, they receive a message related to their risk of relapse—high, moderate, or low. Relapse risk is based on a participant’s EMA responses from the last 7 days (i.e., their substance use in the past 30 min, past week, or more than a week ago). Each level of risk has a corresponding set of messages; one message from the appropriate set is randomly selected and sent to the participant after the EMA is completed. For example, a participant with a low risk of relapse receives a randomly selected message from the set of low-risk messages. Below this risk-adjusted message, participants always see the menu of EMIs.

### Setting, recruitment, and training procedures

Research staff are screening and recruiting participants from two treatment agencies in Chicago (Family Guidance, Haymarket), as well as providing all interventions, follow-up and assessments described here. During the intake process for treatment, staff members give study candidates an overview of the study and an informational flyer and then complete a brief screen to determine eligibility. Individuals who are eligible and agree to participate sign a Consent Form agreeing to be contacted by research staff. Once individuals complete three outpatient sessions or 3 days of residential treatment, researchers contact study candidates to verify contact information and inform candidates that once they are discharged from treatment, they qualify for the next eligibility phase and research staff will be in contact again.

Once candidates are discharged from treatment for any reason or have not attended an outpatient session in 4 weeks, which researchers verify through treatment records, eligible candidates are scheduled for study training. Because smartphones are not allowed on residential units, participants in residential treatment become eligible for training either upon discharge or termination from treatment.

At the beginning of the training session, researchers complete the informed consent process to enroll patients, administer the Global Appraisal of Individual Needs – Quick version 3 (GAIN-Quick 3; discussed further below), and collect locator data and a urine specimen. Next, participants are randomly assigned to one of the four conditions. All individuals participate in relapse-prevention training, after which participants in the control condition leave; those in the other three conditions participate in a smartphone training to ensure they can operate the phone. Next, individuals in the EMA-only and EMA + EMI conditions participate in EMA training; participants in the EMI-only condition receive the EMI training.

**Fig. 1:**
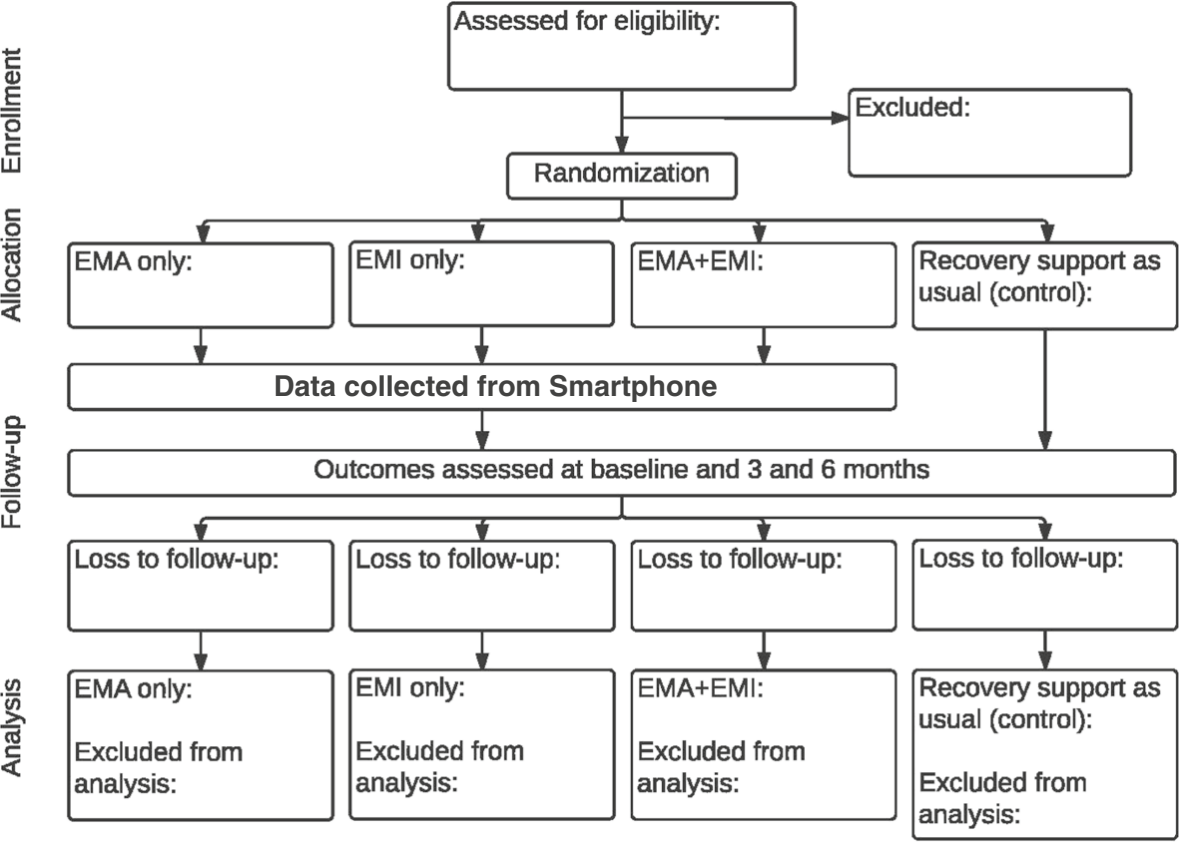
Consolidated Standards of Reporting Trials (CONSORT) diagram of participant flow

Figure 1 shows the flow of participants through the trial.

### Randomization

Participants are randomly assigned to receive recovery support as usual (control), EMAs only, EMIs only, or combined EMAs + EMIs. Randomization uses urn randomization [62] with a base rate of 25% per condition stratified by participant characteristics (gender, race, age, recruitment cohort, level of comfort using a smartphone), pretreatment measures of the dependent variables (days of abstinence, HIV risk behaviors), and prerandomization treatment (level of care, length of stay, type of discharge) to increase the likelihood that participants are distributed similarly across conditions. Urn randomization adjusts the probability of assignment to a condition in ways that simultaneously minimize differences in multiple stratification variables [63, 64]. Only the research coordinator and protocol monitor responsible for scheduling, proficiency testing, and protocol supervision have access to information about who is in each condition. The research coordinator informs participants of their group assignment, describes the next steps, schedules training, and provides an appointment card for the next office visit.

### Measures

Table 2 shows the key measures used in the study, which are described below.

**Table 2:** Summary of key measures

Outcome	Instrument	Frequency
Primary outcome (condensed hypothesis)		
Days of abstinence (greater abstinence among those with EMA, EMI, and EMA + EMI)	Self-report of any alcohol or drug use in last 90 days collected through Global Appraisal of Individual Needs Version 3 (GAIN-Q3) [53].	Baseline and 3 and 6 months after enrollment
	On-site urine drug screens for alcohol, amphetamine/methamphetamine, cannabis, cocaine/benzoylecgonine, and opiates/morphine	Baseline, twice in month 1, and 3 and 6 months after enrollment
Secondary outcomes		
HIV risk behaviors (fewer risk behaviors among those with EMA, EMI, and EMA + EMI)	Self-report of any risk behaviors (needle use, needle sharing, unprotected sex, multiple sexual partners, trading sex for drugs, victimization) in past 90 days collected through GAIN-Q3	Baseline and 3 and 6 months after enrollment
Other outcomes		
EMA and EMI use	Computer logs of EMA responses and EMI use (pages, minutes, interventions, engagement, patterns, comments, and so on)	Continuous

*EMA* Ecological Momentary Assessment, *EMI* Ecological Momentary Intervention

### GAIN-Q3

Researchers trained and supervised by Scott and Dennis interview participants using the 25-min Global Appraisal of Individual Needs Quick Version 3 (GAIN-Q3) [53]. The GAIN-Q3 includes screeners with four to six questions on the recency of symptoms in nine problem areas (school, work, health, stress, HIV risk behaviors/victimization, internalizing disorders, externalizing disorders, substance use disorders, and crime/violence). These screeners were constructed to be correlated at .9 or more with the 11 to 43 symptoms in the full GAIN and cut points with approximately 90% sensitivity, specificity, and area under the curve compared with the full GAIN in the nine areas. Days-of-use measures have been validated against the timeline follow-back [65], collateral [66], records [67], and urine [66–68] and saliva tests [68]. The GAIN-Q3 also includes summary measures for health care utilization, functional impairment, quality of life, and life satisfaction.

### Urine drug screens

Interviews are supplemented with on-site urine screens. Urine will be tested on-site with CLIAA-waived QuikScreen cups using an immunochromatographic assay for rapid (2 to 5 min) qualitative results based on the Substance Abuse and Mental Health Service Authority (SAMHSA)-standard cutoffs for alcohol (20 mg/dl or 0.02% BAC), amphetamine/methamphetamine (1000 ng/ml), cannabis (50 ng/ml), cocaine/benzoylecgonine (300 ng/ml), and opiates/morphine (2000 ng/ml) [69].

### Measurement process/data collection

The team is implementing [70] follow-up procedures, which reliably produce over 90% follow-up rates across studies involving over 40,000 patients regardless of population for follow-up periods ranging from 3 months to 15 years. Steps include: (1) collecting contact information and Consent-to-be-contacted Forms and giving the participant an appointment card, (2) verifying participant contact information, (3) conducting outreach for past-due participants and discussing the situations at weekly research case review meetings, (4) mailing thank-you cards to participants, (5) scheduling follow-up appointments, (6) mailing 3- and 6-week post-enrollment flyers to participants and their collaterals, (7) implementing returned-mail procedures, (8) calling participants 6 weeks before 3- and 6-month research interview appointments to confirm date and location, (9) conducting outreach for unconfirmed cases and reviewing them at weekly meetings, (10) completing follow-up interviews and scheduling next appointments, and (11) implementing a no-show protocol. Progress is being monitored with daily management reports. These procedures help maintain participant contact even for controls and information about whether a phone is lost, stolen, or damaged. Data collected at assessments are identified by study ID, not participant name. The form linking study IDs to names and the data themselves are kept in GAINS ABS, a secure electronic data management system.

### Sample size

For the primary outcome (H1) power analysis, we assumed a sample size of 400 with treatment intake records/services data from the provider (for covariates), interviews by research staff at three time points for the main analysis (baseline and 3 and 6 months), a completion rate of 90 + % per wave of 100 participants, a moderate effect size of Cohen’s *d* = .4 based on Cox’s formula [71] for converting the odds ratio from a pilot study to effect size *d* = LN(1.9)/1.65 = .4)), a target of at least 90% power with a family wise alpha of < .017 on the three a-priori orthogonal contrasts testing the hypothesized effects of EMA, EMI, and their interaction on the primary outcome (days of abstinence), and at least 80% power for all six pairwise comparisons with a family wise alpha of < .0083. The effective *n* for power calculations in repeated measures analysis varies between a lower bound of the number of unique people (*N* = 400 people) and an upper bound of observations from baseline and completed follow-up or 1120 observations (O = (400 people + 90% × 400 people × 2 follow-ups) = 1120) as a function of the Intraclass Correlation Coefficient (ICC) associated with the individual and the number of repeated measures (RM) per person (effective *n*’ = O/(1 + ICC(RM − 1)). In prior samples from the proposed recruitment site [6], the ICC of the days of abstinence between enrollment, 3, and 6 months post enrollment was .52, resulting in an effective *n*’ of 549 (*n*’ = 1120/(1 + .52(3 − 1)) = 549) and 98% power for a two-tailed test of *p* < .017. Note that in the special case of a balanced 2 × 2 as proposed here, the power for the interaction test is the same as each of the main effects [71–74]. In the same study, the ICC of the HIV Risk Behavior Scale was .51, resulting in an effective *n*’ of 554 and 99% power for a two-tailed test of alpha < .05. For the six possible pairwise comparisons, the effective sample size (*n*’ = 275 per pair for H1 and H2) will be sufficient to have 80% power for a two-tailed test of *p* < .0083. Following the recommendations of Piantadosi [75], we have powered this design for the most conservative pairwise tests so that we can look at the efficacy of each combination (EMA, EMI, EMA + EMI) relative to the control and their comparative effectiveness with each other. For mediation analysis (H3), the tests should have 90% + power for a two-tailed test of *p* < .05 for an indirect effect size of *d* = .22 or more.

### Timeline

**Fig. 2:**
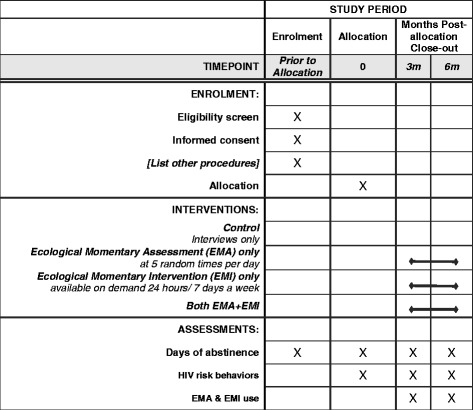
Study schedule of enrolment, intervention, and assessments

Recruitment began in June 2015; the intervention period ends in April 2018. Figure 2 shows the study schedule of enrollment, intervention, and assessments.

### Statistical analysis

#### Missing data

Participants complete their intake and enrollment interviews before randomization. Based on past experience, we expect to complete 90% or more of the 3- and 6-month post-enrollment interviews and have less than 1% missing data on core items. Multiple imputations will be used to replace missing data to allow the least biased estimate for each analysis [76]. To further reduce potential bias, analyses will be rechecked by running them without missing data. If differences result, a general latent variable framework [77] will be used to analyze non-ignorable or systematic missingness that tests whether missing data are qualitatively different by condition.

#### Analysis for aims 1 and 2

We will evaluate the effects of EMAs, EMIs, and EMAs + EMIs on the days of abstinence (H1) and HIV risk behaviors (H2) measured from enrollment to 6 months after enrollment using multilevel structural equation modeling (MSEM) with mixed effects in MPlus (version 6.1). The analysis will model observations (level 1) nested within participants (level 2), with participants modeled as a random factor. Random assignment to the four groups will be modeled as a level 2 predictor in the 2 × 2 factorial design [78] with the three planned orthogonal contrasts shown earlier in Table 1. To be conservative, a Bonferroni correction for family wise error (*p* = 05/3 = 0.017) will be used on primary outcome (days of abstinence) in the three a-priori hypothesis tests for the effect of getting EMA or not (H1a), EMI or not (H1b), and their interaction (H1c). As noted earlier, self-monitoring via EMAs is expected to change behavior. Based on the preliminary studies, we expect EMIs to have an effect in their own right and for the combined EMA + EMI condition to have an additional effect (i.e., an interaction). This and all analyses will use an intent-to-treat approach (i.e., participants are analyzed as assigned regardless of their actual level of participation). Prerandomization participant characteristics, severity, and treatment experience used for urn randomization will be checked for any group differences and used in a propensity score covariate to further reduce unexplained variance. Restricted maximum likelihood estimation (REML) will be used within condition to allow the use of all available data. If there is a significant effect for H1 or H2, we will also examine all six of the possible pairwise comparisons, using an experiment-wise error of .05 and a pairwise test alpha of .05/6 tests = .0083.

#### Mediation analysis (aim 3)

H1 and H2 will provide a test of the direct effects of randomization. To evaluate mediation (H3), we will evaluate (1) the extent to which a direct effect exists between days of abstinence in months 1 to 3 post enrollment and HIV risk behaviors in months 4 to 6 post enrollment (i.e., is the path coefficient significant?) and (2) the extent to which including days of abstinence eliminates or reduces the direct effect of the EMAs, EMIs, and EMAs + EMIs on HIV risk behaviors using the Preacher et al. [79] framework for testing mediation in MSEM with REML in MPlus. Note that to ensure temporal precedence, we have purposely set up random assignment at the time of enrollment, days of abstinence from months 1 to 3 post enrollment, and HIV risk behaviors in months 4 to 6 post enrollment. Loss of statistical significance in the direct path coefficient from the intervention conditions to the HIV risk behaviors would indicate full mediation; a reduction in the path coefficient would indicate partial mediation. The statistical significance of the reduction will be evaluated using MacKinnon’s [80] joint-significance testing of the path *z*-scores, with a Sobel test using a standard error based on bootstrapping and criteria of *p* < .05 on the degree of change. We expect the increased days of abstinence to be the primary mediator of change in HIV risk behaviors, consistent with data from our prior research [6, 7, 81, 82].

#### Combined and additional analyses

Each of the above relationships will be combined to test the overall conceptual model using MSEM with REML in MPlus as part of testing mediation. To better understand for whom the intervention is working, we will also examine whether participant characteristics (including gender, race, age, and familiarity with smartphones), pretreatment days of abstinence and HIV risk behavior, and pre-enrollment treatment experiences moderate any of the observed effects in the combined model using a multigroup MSEM with mixed effects in MPlus.

## Discussion

### Contribution to science and practice

This study will inform the field about whether smartphone EMAs have an independent effect on addiction recovery. It will assess whether EMAs can help patients to monitor multiple risk factors such as people, places, activities, and feelings. It will also provide evidence about whether feedback from EMAs encouraging participant use of EMIs increases the use of EMIs, which ultimately may reduce relapse to substance use and extend recovery. We will also test whether reduced substance use in the EMA + EMI arm leads to fewer HIV risk behaviors. Finally, frequent and regular EMA monitoring of risk of relapse mitigates the biases resulting from retrospective reporting and will likely enhance our understanding of the proximal antecedents of relapse and improve the timeliness and appropriateness of interventions. This information may lead to improved treatment and recovery interventions for people with substance use disorders.

### Challenges

Recruiting prospective participants at treatment initiation but not consenting and enrolling them until discharge from treatment has led to slower recruitment than expected. Many more people than expected are ineligible because they live outside the city of Chicago or have a recovery coach with whom they are been actively involved in the past 30 days.

We are finding in this study, unlike prior studies using only EMIs, that participants make heavy use of streaming video and/or music and this is straining the budget for data contracts. In previous research with smartphone apps, we have had one or two people per study overuse the data plan, but in this study so far, we are finding that nearly half of participants have at least 1 month of very heavy data use. We are analyzing use data to try to identify which apps are resulting in the overages.

### Trial status

The trial has received ethical approval and as of 28 February 2017 we have enrolled 343 participants.

## Additional file

Additional file 1: SPIRIT 2013 Checklist: Recommended items to address in a clinical trial protocol and related documents. (DOC 141 kb)

## Abbreviations

A-CHESS: Addiction – Comprehensive Health Enhancement Support System
EMA: Ecological Momentary Assessment
EMI: Ecological Momentary Intervention
GAIN-Q3: Global Appraisal of Individual Needs Quick Version 3
HIV: Human immunodeficiency virus
RSAU: Recovery support as usual
SUDs: Substance use disorders
