# Supplementary material for: Using smartphones to decrease substance use via self-monitoring and recovery support: study protocol for a randomized control trial
Source: Trials. 2017 Aug 10;18:374. doi: 10.1186/s13063-017-2096-z (PMC5553728; doi:10.1186/s13063-017-2096-z)
Supplement: Additional file 1: — SPIRIT 2013 Checklist: Recommended items to address in a clinical trial protocol and related documents. (DOC 141 kb) [file 13063_2017_2096_MOESM1_ESM.doc]

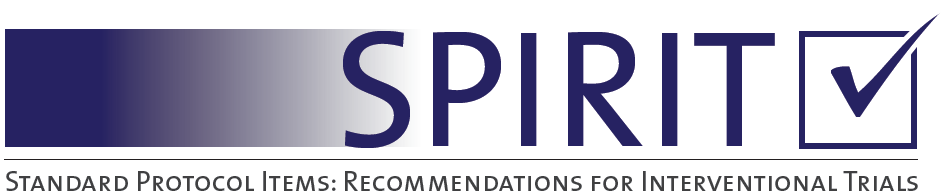


SPIRIT 2013 Checklist: Recommended items to address in a clinical trial protocol and related documents*

| Section/item | Item No | | Description | Addressed on page number |
| --- | --- | --- | --- | --- |
| **We have listed pages in the manuscript that address the points where applicable and highlighted further responses given only here in yellow.**  **Administrative information** | | | |  |
| Title | 1 | Descriptive title identifying the study design, population, interventions, and, if applicable, trial acronym | | ______1_____ |
| Trial registration | 2a | Trial identifier and registry name. If not yet registered, name of intended registry | | ______3_____ |
|  | 2b | All items from the World Health Organization Trial Registration Data Set | | Addendum at the end of this document. |
| Protocol version | 3 | Date and version identifier This protocol is version 1.0, dated June 25, 2015. | | _Here in yellow_ |
| Funding | 4 | Sources and types of financial, material, and other support | | ______19_____ |
| Roles and responsibilities | 5a | Names, affiliations, and roles of protocol contributors | | _____18-19___ |
|  | 5b | Name and contact information for the trial sponsor  **Institute:** National Institute on Drug Abuse (NIDA) grant no. R01DA035879  **Program Official:** Shoshana Y Kahana, **Email**: kahanas@nida.nih.gov, **Phone**: 301-443-2261, **Fax**: 301-443-6814 | | _Here in yellow_ |
|  | 5c | Role of study sponsor and funders, if any, in study design; collection, management, analysis, and interpretation of data; writing of the report; and the decision to submit the report for publication, including whether they will have ultimate authority over any of these activities. The study design, implementation and interpretation are all the responsibility of the authors and do not reflect positions of the government. | | ______19_____ |
|  | 5d | Composition, roles, and responsibilities of the coordinating centre, steering committee, endpoint adjudication committee, data management team, and other individuals or groups overseeing the trial, if applicable (see Item 21a for data monitoring committee)  Christy K. Scott is responsible for the study’s overall scientific direction and implementation. Michael Dennis contributes to the scientific direction and supervises data management and the creation of de-identified data files. David Gustafson contributes to the scientific direction and has overall responsibility for the A-CHESS software used in the study. Scott, Dennis, Gustafson, and McTavish are the steering committee for the study. Savita Prakesh is the independent data safety monitor. | | _Here in yellow__ |
| Introduction |  |  | |  |
| Background and rationale | 6a | Description of research question and justification for undertaking the trial, including summary of relevant studies (published and unpublished) examining benefits and harms for each intervention | | ___4-7_______ |
|  | 6b | Explanation for choice of comparators | | ___7-10_______ |
| Objectives | 7 | Specific objectives or hypotheses | | _____7_______ |
| Trial design | 8 | Description of trial design including type of trial (eg, parallel group, crossover, factorial, single group), allocation ratio, and framework (eg, superiority, equivalence, noninferiority, exploratory) | | _____7_______ |
| Methods: Participants, interventions, and outcomes | | | |  |
| Study setting | 9 | | Description of study settings (eg, community clinic, academic hospital) and list of countries where data will be collected. Reference to where list of study sites can be obtained | _____11______ |
| Eligibility criteria | 10 | | Inclusion and exclusion criteria for participants. If applicable, eligibility criteria for study centres and individuals who will perform the interventions (eg, surgeons, psychotherapists) | ____10-11_____ |
| Interventions | 11a | | Interventions for each group with sufficient detail to allow replication, including how and when they will be administered | ___7-10_______ |
|  | 11b | | Criteria for discontinuing or modifying allocated interventions for a given trial participant (eg, drug dose change in response to harms, participant request, or improving/worsening disease)  We will discontinue a participant who is repeatedly threatening other participants or staff. The standard procedures allow for replaced a lost or damaged phone once; after that we will not typically issue a third phone (defacto ending participation). | _Here in yellow__ |
|  | 11c | | Strategies to improve adherence to intervention protocols, and any procedures for monitoring adherence (eg, drug tablet return, laboratory tests) | ______13______ |
|  | 11d | | Relevant concomitant care and interventions that are permitted or prohibited during the trial | ______6______ |
| Outcomes | 12 | | Primary, secondary, and other outcomes, including the specific measurement variable (eg, systolic blood pressure), analysis metric (eg, change from baseline, final value, time to event), method of aggregation (eg, median, proportion), and time point for each outcome. Explanation of the clinical relevance of chosen efficacy and harm outcomes is strongly recommended | ____Table 2___ |
| Participant timeline | 13 | | Time schedule of enrolment, interventions (including any run-ins and washouts), assessments, and visits for participants. A schematic diagram is highly recommended (see Figure) | ____Figure 2____ |
| Sample size | 14 | | Estimated number of participants needed to achieve study objectives and how it was determined, including clinical and statistical assumptions supporting any sample size calculations | ______14______ |
| Recruitment | 15 | | Strategies for achieving adequate participant enrolment to reach target sample size  Although the start of recruitment was slower than expected (p. 17), we have since caught up and expect to meet our enrolment goal. | __Here in yellow__ |
| **Methods: Assignment of interventions (for controlled trials)** | | | |  |
| Allocation: |  | |  |  |
| Sequence generation | 16a | | Method of generating the allocation sequence (eg, computer-generated random numbers), and list of any factors for stratification. To reduce predictability of a random sequence, details of any planned restriction (eg, blocking) should be provided in a separate document that is unavailable to those who enrol participants or assign interventions | _____11-12____ |
| Allocation concealment mechanism | 16b | | Mechanism of implementing the allocation sequence (eg, central telephone; sequentially numbered, opaque, sealed envelopes), describing any steps to conceal the sequence until interventions are assigned | _____12______ |
| Implementation | 16c | | Who will generate the allocation sequence, who will enrol participants, and who will assign participants to interventions . | _____12______ |
| Blinding (masking) | 17a | | Who will be blinded after assignment to interventions (eg, trial participants, care providers, outcome assessors, data analysts), and how Is there any blinding? The study is not blinded. | _____12____ |
|  | 17b | | If blinded, circumstances under which unblinding is permissible, and procedure for revealing a participant’s allocated intervention during the trial. Not applicable. | _____12_____ |
| **Methods: Data collection, management, and analysis** | | | |  |
| Data collection methods | 18a | | Plans for assessment and collection of outcome, baseline, and other trial data, including any related processes to promote data quality (eg, duplicate measurements, training of assessors) and a description of study instruments (eg, questionnaires, laboratory tests) along with their reliability and validity, if known. Reference to where data collection forms can be found, if not in the protocol | ____12-14____ |
|  | 18b | | Plans to promote participant retention and complete follow-up, including list of any outcome data to be collected for participants who discontinue or deviate from intervention protocols | _____13_____ |
| Data management | 19 | | Plans for data entry, coding, security, and storage, including any related processes to promote data quality (eg, double data entry; range checks for data values). Reference to where details of data management procedures can be found, if not in the protocol Further details available in IRB & DSMB plans. | _____13-14_____ |
| Statistical methods | 20a | | Statistical methods for analysing primary and secondary outcomes. Reference to where other details of the statistical analysis plan can be found, if not in the protocol. Further details on ClinicalTrials.gov , **Identifier:**  NCT02132481. | ___15-16_____ |
|  | 20b | | Methods for any additional analyses (eg, subgroup and adjusted analyses) | _____16-17_____ |
|  | 20c | | Definition of analysis population relating to protocol non-adherence (eg, as randomised analysis), and any statistical methods to handle missing data (eg, multiple imputation) | ______16_____ |
| **Methods: Monitoring** | | | |  |
| Data monitoring | 21a | | Composition of data monitoring committee (DMC); summary of its role and reporting structure; statement of whether it is independent from the sponsor and competing interests; and reference to where further details about its charter can be found, if not in the protocol. Alternatively, an explanation of why a DMC is not needed  Savita Prakesh serves as the independent data safety monitor. The DSM functions independently of the sponsor of the study, the PI and research team, and competing interests. During the year, the DSM is provided a report on any serious adverse events (SAE) within 2 business days of their occurrence and consulted on any changes to the status of an individual or the protocol. Annually (after IRB review and before progress report is due to NIDA), the research team prepare a report about the progress of the study for the DSM. A copy of this report and all SAE and unexpected adverse event (AE) reports during the year are then sent to the DSM. After the research time answers any questions from the DSM about the trial progress and issues that have copy up, the DSM provides the research team with a letter approving the continuation of the study. | __Here in yellow._ |
|  | 21b | | Description of any interim analyses and stopping guidelines, including who will have access to these interim results and make the final decision to terminate the trial  Comparison of the 4 groups by the main and secondary outcomes are done once a year for the DSM report described above. Access to this report is limited to the PI, steering committee and funder. | __Here in yellow._ |
| Harms | 22 | | Plans for collecting, assessing, reporting, and managing solicited and spontaneously reported adverse events and other unintended effects of trial interventions or trial conduct.  In accordance with federal law and Chestnut Health Systems regulations, any human subjects issues that arise during the trial will be reported to the IRB. These issues are also summarized and included in the annual progress report. | __Here in yellow._ |
| Auditing | 23 | | Frequency and procedures for auditing trial conduct, if any, and whether the process will be independent from investigators and the sponsor.  Management reports on recruitment, randomization, implementation and follow-up are generated weekly and distributed to the study’s steering committee and key staff. As noted in the response to item 21a, the trial is subjected to an annual review by the independent Data Monitoring Committee. In addition, we have access to this committee throughout the year if the need arises. Preliminary data from the mobile software system was checked against expectations during the first 6 months until it met expectations and has been rechecked annually since then. Scale psychometrics and validation against urine are checked annual as part of the above progress report. | __Here in yellow._ |
| Ethics and dissemination | | | |  |
| Research ethics approval | 24 | | Plans for seeking research ethics committee/institutional review board (REC/IRB) approval | _____10______ |
| Protocol amendments | 25 | | Plans for communicating important protocol modifications (eg, changes to eligibility criteria, outcomes, analyses) to relevant parties (eg, investigators, REC/IRBs, trial participants, trial registries, journals, regulators) Any changes will be submitted to and approved by the IRB and DSM. They will also be reported to the project officer as part of the annual progress report. | __Here in yellow._ |
| Consent or assent | 26a | | Who will obtain informed consent or assent from potential trial participants or authorised surrogates, and how (see Item 32) | _____11______ |
|  | 26b | | Additional consent provisions for collection and use of participant data and biological specimens in ancillary studies, if applicable | _Does not apply._ |
| Confidentiality | 27 | | How personal information about potential and enrolled participants will be collected, shared, and maintained in order to protect confidentiality before, during, and after the trial | ____13-14______ |
| Declaration of interests | 28 | | Financial and other competing interests for principal investigators for the overall trial and each study site | ____18_______ |
| Access to data | 29 | | Statement of who will have access to the final trial dataset, and disclosure of contractual agreements that limit such access for investigators  Scott, Dennis, and Gustafson will have access to the final dataset. | __Here in yellow._ |
| Ancillary and post-trial care | 30 | | Provisions, if any, for ancillary and post-trial care, and for compensation to those who suffer harm from trial participation. None are planned. | __Here in yellow._ |
| Dissemination policy | 31a | | Plans for investigators and sponsor to communicate trial results to participants, healthcare professionals, the public, and other relevant groups (eg, via publication, reporting in results databases, or other data sharing arrangements), including any publication restrictions. We plan to disseminate results of the trial through publication, without restrictions, regardless of the direction or magnitude of its effects. | __Here in yellow._ |
|  | 31b | | Authorship eligibility guidelines and any intended use of professional writers. The steering committee includes the core authors and has no plans to use professional writers. | __Here in yellow._ |
|  | 31c | | Plans, if any, for granting public access to the full protocol, participant-level dataset, and statistical code Once we have completed our analysis, we will make the data available to researchers who contact us and provide evidence that their research has been approved by appropriate review bodies. | __Here in yellow._ |
| Appendices |  | |  |  |
| Informed consent materials | 32 | | Model consent form and other related documentation given to participants and authorised surrogates  The consent forms are submitted with the manuscript as separate files. | __Here in yellow._ |
| Biological specimens | 33 | | Plans for collection, laboratory evaluation, and storage of biological specimens for genetic or molecular analysis in the current trial and for future use in ancillary studies, if applicable | _Does not apply._ |

*It is strongly recommended that this checklist be read in conjunction with the SPIRIT 2013 Explanation & Elaboration for important clarification on the items. Amendments to the protocol should be tracked and dated. The SPIRIT checklist is copyrighted by the SPIRIT Group under the Creative Commons “[Attribution-NonCommercial-NoDerivs 3.0 Unported](http://www.creativecommons.org/licenses/by-nc-nd/3.0/)” license.

**Addendum:**

**WHO Checklist:**

1. Primary registry and trial identifying number: ClinicalTrials.govNCT02132481

2. Date of registration in primary registry: 5/5/14

3. Secondary identifying numbers: National Institute on Drug Abuse (NIDA) grant no. R01 DA021174.

4. Source(s) of monetary or material support: National Institute on Drug Abuse (NIDA)

5. Primary sponsor: National Institute on Drug Abuse (NIDA)

6. Secondary sponsor(s): None

7. Contact for public queries: Christy K. Scott, PhD, at 312-664-4321 or [cscott@chestnut.org](mailto:cscott@chestnut.org)

8. Contact for scientific queries: Christy K. Scott, PhD, at 312-664-4321 or [cscott@chestnut.org](mailto:cscott@chestnut.org)

9. Public title: Using Smartphones to Provide Recovery Support Services (SRSS) / aka Project EMA

10: Scientific title: : Using Smartphones to Provide Recovery Support Services (SRSS) / aka Project EMA

11. Countries of recruitment: U.S.A.

12. Health condition(s) or problem(s) studied: Substance Use Disorders (SUD)

13: Intervention(s):

The study tests elements of a smartphone application designed to improve recovery from substance use disorders and reduce HIV risk behaviors. Patients leaving substance use treatment will be assigned to one of four groups for six months. Three groups have the smartphone app and one functions as the control. All groups receive recovery support as usual (RSAU). . The 4 groups are:

- One group has the smartphone app with Ecological Momentary Assessments (EMAs), which ask patients at five random times per day about people, places, activities, and moods that make them want to use substances, support their recovery, or have no impact. The goal is to improve patient’s self-monitoring.
- The second group has the smartphone app with Ecological Momentary Interventions (EMIs), which provide information about addiction and recovery, reminders of motivators and coping mechanisms, a calendar of healthy activities, distractions, and social support from peers.
- The third group has the smartphone app with both EMAs and EMIs. EMIs are delivered to the participant when EMA responses indicate risk.
- The control group has RSAU only, which involves giving patients a recovery plan and relevant community referrals upon discharge from treatment.

14. Key inclusion and exclusion criteria:

Men and women are eligible if they: (a) are 18 years old or older, (b) met criteria for SUDs in the year prior to treatment intake, (c) currently live in Chicago, (d) communicate in English, and (e) are cognitively able to provide informed consent. Individuals are ineligible if they: (a) currently live outside Chicago or plan to live outside of Chicago during the six months of the study, (b) expect to be in jail, prison, or another setting that would prevent the use of smartphones, (c) are unable to use a smartphone because of disability or health condition, (d) are unwilling to learn to use a smartphone or to complete a survey using a smartphone, (e) are admitted to a treatment program that provides intensive services post-discharge, (f) have a recovery coach and have been in contact with the recovery coach in the last 30 days (g) fail the cognitive test, and (h) have ever been diagnosed with or told by a doctor that they have schizophrenia and/or are bi-polar.

15. Study type:

Randomized control trial, 2 x 2 factorial design, designed to test the effect of Ecological Momentary Assessments, Ecological Momentary Interventions, and Ecological Momentary Assessments + Ecological Momentary Interventions vs. recovery support as usual on days of abstinence and, secondarily, on HIV risk behaviors

16. Date of first enrollment: 6/23/15

17. Target sample size: 400 participants (100 per condition)

18. Recruitment status: Recruiting: participants are currently being recruited and enrolled

19. Primary outcome:

Days of abstinence from alcohol or other drugs, measured by self-report collected through the Global Appraisal of Individual Needs Version 3 at baseline and months 3 and 6 after enrollment

20. Key secondary outcomes:

- HIV risk behaviors, measured by self-report collected through the Global Appraisal of Individual Needs Version 3 at baseline and months 3 and 6 after enrollment
- Abstinence at 3 months will mediate effects at 6 months of Ecological Momentary Assessments, Ecological Momentary Interventions, and Ecological Momentary Assessments + Ecological Momentary Interventions on HIV risk behaviors, measured by self-report collected through the Global Appraisal of Individual Needs Version 3 at 3 and 6 months
